# Prognostic value of measurable residual disease monitoring by next-generation sequencing before and after allogeneic hematopoietic cell transplantation in acute myeloid leukemia

**DOI:** 10.1038/s41408-021-00500-9

**Published:** 2021-06-04

**Authors:** Hee-Je Kim, Yonggoo Kim, Dain Kang, Hoon Seok Kim, Jong-Mi Lee, Myungshin Kim, Byung-Sik Cho

**Affiliations:** 1 Department of Hematology, Catholic Hematology Hospital, Seoul St. Mary’s Hospital, College of Medicine, The Catholic University of Korea, Seoul, Republic of Korea; 2 Leukemia Research Institute, College of Medicine, The Catholic University of Korea, Seoul, Republic of Korea; 3 Catholic Genetic Laboratory Center, Seoul St. Mary’s Hospital, College of Medicine, The Catholic University of Korea, Seoul, Republic of Korea; 4 Department of Laboratory Medicine, College of Medicine, The Catholic University of Korea, Seoul, Republic of Korea

**Keywords:** Cancer genomics, Acute myeloid leukaemia, Translational research, Acute myeloid leukaemia

## Abstract

Given limited studies on next-generation sequencing-based measurable residual disease (NGS-MRD) in acute myeloid leukemia (AML) patients after allogeneic hematopoietic stem cell transplantation (allo-HSCT), we longitudinally collected samples before and after allo-HSCT from two independent prospective cohorts (*n* = 132) and investigated the prognostic impact of amplicon-based NGS assessment. Persistent mutations were detected pre-HSCT (43%) and 1 month after HSCT (post-HSCT-1m, 20%). All persistent mutations at both pre-HSCT and post-HSCT-1m were significantly associated with post-transplant relapse and worse overall survival. Changes in MRD status from pre-HSCT to post-HSCT-1m indicated a higher risk for relapse and death. Isolated detectable mutations in genes associated with clonal hematopoiesis were also significant predictors of post-transplant relapse. The optimal time point of NGS-MRD assessment depended on the conditioning intensity (pre-HSCT for myeloablative conditioning and post-HSCT-1m for reduced-intensity conditioning). Serial NGS-MRD monitoring revealed that most residual clones at both pre-HSCT and post-HSCT-1m in patients who never relapsed disappeared after allo-HSCT. Reappearance of mutant clones before overt relapse was detected by the NGS-MRD assay. Taken together, NGS-MRD detection has a prognostic value at both pre-HSCT and post-HSCT-1m, regardless of the mutation type, depending on the conditioning intensity. Serial NGS-MRD monitoring was feasible to compensate for the limited performance of the NGS-MRD assay.

## Introduction

Disease relapse remains the major cause of treatment failure in acute myeloid leukemia (AML) treated with allogeneic hematopoietic stem cell transplantation (allo-HSCT)^1^. Measurable residual disease (MRD) detection is highly valuable in predicting relapse and survival in AML patients in complete remission (CR)^2,3^. However, real-time quantitative polymerase chain reaction-based detection has limited applicability for some targets, and there are difficulties in the standardization of multiparametric flow cytometry. Thus, novel means of detecting MRD that can be standardized and applied to all AML patients are needed. In this context, next-generation sequencing (NGS) enables reliable detection of patient-specific mutations covering complete genes at both the time of diagnosis and CR^4^.

However, recent studies have demonstrated not only the consistent prognostic value of NGS-based MRD (NGS-MRD) detection, but also its limitations related to its sensitivity and specificity and its inability to correctly discriminate between residual leukemia and clonal hematopoiesis^5–13^. In earlier studies, NGS-MRD assessments were performed after high-dose induction treatment, which may be a suitable approach for selecting the appropriate consolidation treatment^8,9,12^. A few studies have shown that NGS-MRD detection in the setting of allo-HSCT can help predict clinical outcomes^7,10,11,13,14^. However, the NGS-MRD assessments in those studies were generally only performed pre-transplantation, and no NGS-MRD data after transplantation were collected except for one study^10^. Moreover, the results were discordant, possibly due to differences in cohorts, sample sources (bone marrow (BM) or peripheral blood (PB)), NGS techniques, the definition of MRD positivity, and strategies for mutations related to clonal hematopoiesis (i.e., clonal hematopoiesis of indeterminate potential (CHIP)). These mutations include: *DNMT3A*; *TET2*; *ASXL1* (DTA); *IDH2*; *IDH1*; and other less prevalent mutations, such as *JAK2*, *CBL*, *SRSF2*, and *SF3B1*^7,11,13,14^. Thus, the prognostic value of NGS-MRD in the setting of allo-HSCT in AML is yet to be fully elucidated. In addition, the clinical impact of dynamic changes in persistent mutations before and after allo-HSCT has not been clarified to date. The influence of different transplant strategies, such as that of the conditioning intensity, also needs to be properly evaluated in prospective studies.

Thus, this study aimed to investigate the role of NGS-MRD detection in the setting of allo-HSCT to ultimately elucidate the optimal time points, cutoff values, candidates, role of DTA or CHIP, and influence of transplant strategy. Towards this goal, we collected samples and clinical data from two independent prospective cohorts and longitudinally tracked clonal changes before and after allo-HSCT.

## Materials and methods

### Study design and patients

This study evaluated 146 patients with AML who underwent allo-HSCT at CR in two independent prospective cohorts in the Catholic Hematology Hospital between 2013 and 2018. The inclusion criteria were age over 19 years and availability of BM DNA both at diagnosis and in CR before allo-HSCT. Cohort 1 included patients who received transplants from matched unrelated donors and haploidentical familial donors. Cohort 2 included patients who received transplants from similar donors to those in Cohort 1 plus transplants from matched sibling donors. The patient inclusion process is shown in Supplementary Fig. S1.

Samples were obtained at three time points: (1) the time of diagnosis, pre-HSCT (before conditioning therapy, median: 27 days before transplantation, range: 10–42 days), (2) post-HSCT (1, 3, and 6 months and yearly thereafter), and (3) at relapse. Among the 132 (90%) patients who had somatic mutations at diagnosis, 114 (86%) had available BM DNA at 1 month after allo-HSCT (post-HSCT-1m, median: 28 days after transplantation, range: 26–30 days). Cohort 1 had a higher incidence of CR2, and Cohort 2 included more elderly patients. No other pre-transplant characteristics significantly differed between the two cohorts (Supplementary Table S1). The treatment courses and transplantation procedures were performed as previously described^15^. The Institutional Review Board of the Catholic Medical Center approved the current study. Informed consent was obtained from all subjects and all analyses were performed according to the Institutional Review Board guidelines and the tenets of the Declaration of Helsinki. Cohorts 1 and 2 were registered at ClinicalTrials.gov (#NCT01751997) and the Clinical Research Information Service (#KCT0002261), respectively.

### NGS-MRD detection

NGS analysis was performed using St. Mary’s customized NGS panel for acute leukemia, i.e., the “SM Acute leukemia panel.” Ion AmpliSeq Technology (Thermo Fisher Scientific) was used to amplify 67 genes (Supplementary Table S2) using an Ion Chef™ system (Thermo Fisher Scientific) and an Ion S5 XL Sequencer (Thermo Fisher Scientific)^16^.

Annotated variants were classified into four tiers according to the Standards and Guidelines of the Association for Molecular Pathology^17^. Bioinformatics analysis was carried out using both customized and manufacturer-provided pipelines. Variants were selected and annotated using analytics algorithms and public databases^18^. Subsequently, trackable somatic mutations specific to each patient were selected. For NGS-MRD, we carefully inspected the mutations and determined the residual variant allele fraction (% VAF), which was calculated by dividing the number of mutant sequencing reads with the number of total sequencing reads. All mutations were manually verified using the Integrative Genomic Viewer^19^. Across all time points, the mean of on-target reads, depth of on-target regions, and uniformity were 99.4%, 2406×, and 96.9%, respectively. Details of the quality control matrices are summarized in Supplementary Table S3.

### Statistical analysis

Categorical variables were compared using Chi-square test or Fisher’s exact test while continuous variables were analyzed with the Mann-Whitney *U* test. Overall survival (OS) and disease-free survival (DFS) curves were plotted using the Kaplan-Meier method and analyzed with the log-rank test. Cumulative incidence was used to estimate the probability of cumulative incidence of relapse (CIR) and non-relapse mortality (NRM), to treat non-relapse death and relapse as competing risk factors for relapse and NRM, respectively. Cumulative incidence was compared across groups using the Gray test. Results were expressed as the hazard ratio with a 95% confidence interval (95% CI). For multivariate analysis, variables with a *p*-value of <0.10 in the univariate analysis were entered into a Cox proportional hazards model or proportional hazards model for a sub-distribution of competing risk factors. A detailed description is provided in the Supplementary materials. All statistical analyses were performed using SPSS, version 13.0 (SPSS, Inc., Chicago, IL) and R-software (version 3.4.1, R Foundation for Statistical Computing, 2017).

## Results

### Patient characteristics

Overall, 132 pre-HSCT and 114 post-HSCT-1m patients underwent NGS-MRD. Persistent mutations were detectable in 43% and 20% of pre-HSCT and post-HSCT-1m samples, respectively. Table 1 describes the characteristics of the patients with or without persistent mutations at each time point. Persistent pre-HSCT mutations were more frequent in Cohort 2 than in Cohort 1, whereas there was no significant between-group difference in the rate of persistent post-HSCT-1m mutations. No significant differences in patient characteristics were observed according to the presence of persistent mutations at each time point, except for older age at pre-HSCT and a greater incidence of CR2 at post-HSCT-1m in patients with persistent mutations.

**Table 1:** Comparison of characteristics between patients with and without persistent mutations at each time point.

Variables	Pre-HSCT (*n* = 132)			Post-HSCT-1m (*n* = 114)		
	No persistent mutations (*n* = 75)	Persistent mutations (*n* = 57)	*p-*Value	No persistent mutations (*n* = 91)	Persistent mutations (*n* = 23)	*p-*Value
Cohort			0.029			0.147
Cohort #1	42 (56)	21 (37)		40 (44)	14 (61)	
Cohort #2	33 (44)	36 (63)		51 (56)	9 (39)	
Age at HSCT, years			0.029			0.341
Median (range)	45 (19–74)	54 (19–70)		48 (19–74)	55 (21–69)	
Age group, *n* (%)			0.589			0.217
<60 years	62 (83)	45 (79)		72 (81)	16 (70)	
≥60 years	13 (17)	12 (21)		17 (19)	7 (30)	
Sex, *n* (%)			0.349			0.319
Male	43 (57)	28 (49)		54 (59)	11 (48)	
Female	32 (43)	29 (51)		37 (41)	12 (52)	
AML type, *n* (%)			0.365			0.364
De novo	68 (91)	47 (93)		77 (85)	22 (96)	
Secondary	6 (8)	9 (16)		12 (13)	1 (4)	
Therapy-related	1 (1)	1 (2)		2 (2)	0	
WBC count, at diagnosis			0.520			0.806
Median (range)	11.3 (0.5–226.2)	13.2 (0.6–188.7)		11.6 (0.5–266.2)	15.4 (0.6–188.7)	
WBC group, at diagnosis, *n* (%)			0.090			0.464
<50 × 10^9^/L	62 (83)	40 (70)		70 (77)	16 (70)	
≥50 × 10^9^/L	13 (17)	17 (30)		21 (23)	7 (30)	
Cytogenetic risk group, *n* (%)^a^			0.147			0.408
Favorable	16 (21)	5 (9)		14 (15)	5 (22)	
Intermediate	46 (53)	41 (72)		59 (65)	16 (70)	
Adverse	13 (17)	11 (19)		18 (20)	2 (8)	
2017 ELN risk group, *n* (%)			0.705			0.524
Favorable	28 (37)	19 (33)		31 (34)	10 (43)	
Intermediate	27 (36)	19 (33)		35 (38)	6 (26)	
Adverse	20 (27)	19 (33)		25 (28)	7 (30)	
Disease status at HSCT, *n* (%)			0.315			0.025
CR1	74 (99)	54 (95)		90 (99)	20 (87)	
CR2	1 (1)	3 (5)		1 (1)	3 (13)	
Donor type, *n* (%)			0.189			0.943
Matched sibling	12 (16)	15 (26)		17 (18)	4 (17)	
Matched unrelated	34 (45)	18 (32)		36 (40)	10 (44)	
Haploidentical	29 (39)	24 (42)		38 (42)	9 (39)	
Relationship, *n* (%)			0.109			0.732
Related	41 (55)	39 (68)		55 (60)	13 (57)	
Unrelated	34 (45)	18 (32)		36 (40)	10 (43)	
Stem cell source, *n* (%)			0.633			-
Peripheral blood	72 (96)	56 (98)		91 (100)	23 (100)	
Bone marrow	3 (4)	1 (2)		0	0	
HLA disparity, *n* (%)			0.690			0.819
Full matched	46 (61)	33 (58)		53 (58)	14 (61)	
Mismatch	29 (39)	24 (42)		38 (42)	9 (39)	
Conditioning intensity, *n* (%)			0.281			0.119
Myeloablative	36 (48)	22 (39)		40 (44)	6 (26)	
Reduced-toxicity	39 (52)	35 (61)		51 (56)	17 (74)	
GVHD prophylaxis, *n* (%)			0.146			1.000
Cyclosporine + MTX	12 (16)	15 (26)		17 (19)	4 (17)	
Tacrolimus + MTX	63 (84)	42 (74)		74 (81)	19 (83)	
ATG, total dose, *n* (%)			0.587			0.184
Not used	11 (15)	5 (9)		10 (11)	1 (4)	
2.5 mg/kg	29 (39)	24 (42)		33 (36)	13 (57)	
5.0 mg/kg	35 (47)	28 (49)		48 (53)	9 (39)	
HCT-CI at HSCT, *n* (%)			0.911			0.394
0–2	52 (69)	39 (68)		63 (69)	18 (78)	
>2	23 (31)	18 (32)		28 (31)	5 (22)	
Sex match, *n* (%)			0.608			0.071
Female to male	13 (17)	8 (14)		19 (21)	1 (4)	
Others	62 (83)	49 (86)		72 (79)	22 (96)	

*AML* acute myeloid leukemia, *ATG* anti-thymocyte globulin, *CR1* first complete remission, *CR2* second complete remission, *ELN* European LeukemiaNet, *GVHD* graft-versus-host disease, *HCT-CI* hematopoietic cell transplant-comorbidity index, *HSCT* hematopoietic stem cell transplantation, *MTX* methotrexate, *n* number, *WBC* white blood cells.

^a^Cytogenetic risk group was defined by refinement of cytogenetic classification by the United Kingdom Medical Research Council trials (Grimwade, et al.^24^).

### Landscape of somatic mutations and dynamic changes in allelic burden at diagnosis and during the peri-transplant period

**Fig. 1:**
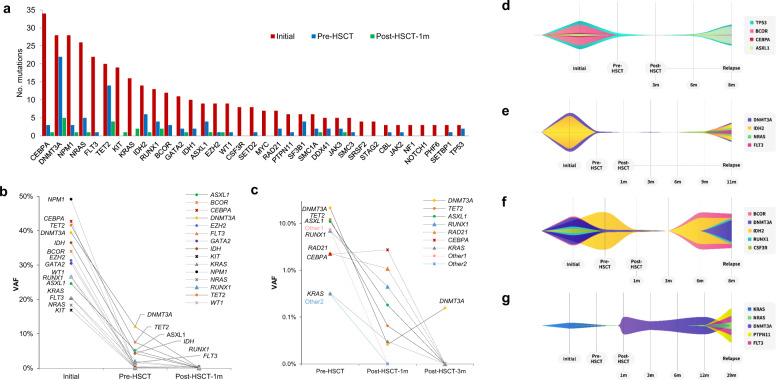
Mutational dynamics of the 132 AML patients. **a** Bar plot showing the mutational status of genes at initial diagnosis (red bar), before transplantation (pre-HSCT, blue bar), and 1 month after transplantation (post-HSCT-1m, green bar). **b** Clonal dynamics of mutations from initial diagnosis to pre-HSCT to post-HSCT-1m. Each symbol represents the mean VAF. **c** Changes in the mean VAF in patients with NGS-MRD positivity without relapse. Many mutations showed late clearance at post-HSCT-3m. Other1 shows the mean VAF of genes including *CBL*, *IDH*, *NPM1*, *SETBP1*, *SF3B1*, and *TP53*. Other2 shows the mean VAF of genes including *BCOR*, *BRAF*, *DDX41*, *FBXW7*, *GATA2*, *NRAS*, and *SETD2*. **d**–**g** Variant allele frequency dynamics with mutational clearance and evolution at initial diagnosis and during follow-up. **d**–**g** Fish plots showing the appearance of mutations before overt relapse. **d** Selective clearance of the *CEBPA* mutation. **e** Evolution of new *NRAS* and *FLT3* mutations at relapse. **f** Later clearance of *DNMT3A* and *IDH2* mutations, and reappearance of *BCOR* and *IDH2* mutations before overt relapse. **g** A donor organ-originated *DNMT3A* mutation detected post-HSCT-1m that diminished at relapse, with evolving *NRAS, PTPN11*, and *FLT3* mutations at relapse.

The genetic landscapes of all patients are shown in Fig. 1a and Supplementary Fig. S2. We detected a total of 389 somatic mutations in 47 genes of the 132 patients, with a median of 3 mutations (interquartile range, IQR: 2–4 mutations) per patient. The most common somatic mutation was in *CEBPA*, followed by that in *DNMT3A*, *NPM1*, and *NRAS*. The median VAF of mutations in the initial samples was 34.39% (IQR: 10.80–45.87%). In paired pre-HSCT samples, we detected 97 mutations in 57 patients, including 90 mutations detected in initial samples and 7 CR-specific mutations not present in initial samples. The median VAF of mutations in the pre-HSCT samples was 2.69% (IQR: 0.38–16.36%). We next analyzed post-HSCT-1m mutations and detected 26 mutations in 23 patients, with the most common being mutations in *DNMT3A* and *TET2*. The median VAF of mutations in the post-HSCT-1m samples was 0.19% (IQR: 0.13–0.60%). We observed a significant reduction in VAF from diagnosis, pre-HSCT, to post-HSCT-1m (Fig. 1b). Particularly, allo-HSCT had a significant impact on the remaining pre-HSCT mutations, clearing additional *DNMT3A* (16/19, 84%), *TET2* (13/14, 71%), and *ASXL1* (2/2,100%) mutations. Summaries of the MRD clearance rate of each mutation are shown in Supplementary Table S4. By molecular pathway, chromatin/cohesion, DNA methylation, and RNA splicing had lower mutational clearance at pre-HSCT. They were further cleared (over 80% clearance) at post-HSCT-1m.

### Prognostic value of NGS-MRD detection

**Fig. 2:**
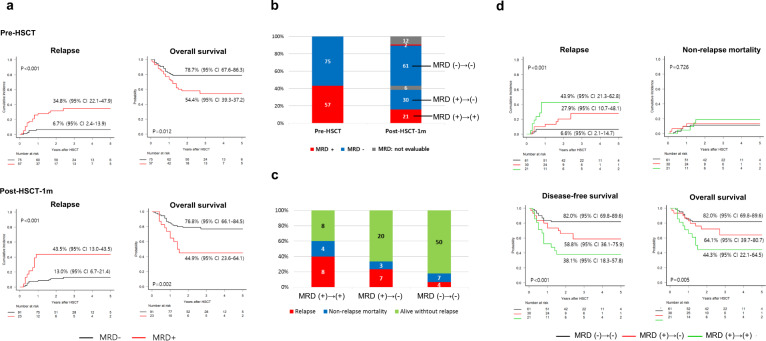
Prognostic roles of NGS-MRD at pre-HSCT and post-HSCT. **a** Cumulative incidence of relapse and overall survival according to NGS-MRD status at pre-HSCT and post-HSCT-1m. **b**–**d** Prognostic effect of changes in NGS-MRD status between pre-HSCT and post-HSCT-1m. **b** NGS-MRD status at pre-HSCT and post-HSCT-1m. **c** Outcomes in the three groups classified by changes in NGS-MRD status between pre-HSCT and post-HSCT-1m. **d** Survival outcomes in the three groups classified by changes in NGS-MRD status between pre-HSCT and post-HSCT-1m.

With a median follow-up duration of 33 months for survivors, the overall CIR was 18.7% (95% CI: 12.4–26.0). Cohorts 1 and 2 had a CIR of 15.9% (95% CI: 8.1–26.0) and 22.5% (95% CI: 12.4–34.4), respectively. Patients with persistent mutations were at significantly greater risk of relapse than those without persistent mutations (pre-HSCT: 34.8% vs. 6.7%, *p* < 0.001; post-HSCT-1m: 43.5% vs. 12.3%, *p* < 0.001), resulting in inferior OS (Fig. 2a and Supplementary Table S5). NGS-MRD detection also had a significant predictive value for CIR and OS at each time point in each cohort (Supplementary Fig. S3). To determine the optimal VAF threshold for predicting post-transplant relapse, we compared various cutoffs for VAF (0%, 0.2%, 1.0%, 2.0%, 2.5%, and 5.0%) and found that 0% VAF resulted in the most effective positive and negative predictive values (Supplementary Fig. S4 and Supplementary Table S6). Thereafter, NGS-MRD positivity defined by a failure of complete clearance of mutations (VAF cutoff of 0%) was independently associated with increased CIR and worse survival in the multivariate analysis at each time point (Table 2).

**Table 2:** Multivariate analysis for factors affecting survival outcomes.

Model #1 (*n* = 132)	*n*	Relapse		Non-relapse mortality		Disease-free survival		Overall survival	
		HR (95% CI)	*p*- Value	HR (95% CI)	*p*-Value	HR (95% CI)	*p*-Value	HR (95% CI)	*p*-Value
NGS-MRD at pre-HSCT^a^									
Negative	75	1				1		1	
Positive	57	5.59 (2.07–15.12)	0.001			2.26 (1.37–4.77)	0.003	2.07 (1.08–3.95)	0.027
Disease state									
CR1	128	1				1		1	
CR2	4	2.57 (0.59–11.16)	0.207			2.60 (0.79–8.60)	0.116	3.48 (1.02–11.49)	0.046
Cohort									
Cohort #1	63			1					
Cohort #2	69			0.42 (0.14–1.25)	0.119				
Relationship									
Related	80			1					
Unrelated	52			2.47 (0.92–6.58)	0.072				
Sex match									
Female to male	21			1					
Others	111			0.33 (0.12–0.85)	0.022				

Model #2 (*n* = 114)	*n*	Relapse		Non-relapse mortality		Disease-free survival		Overall survival	
		HR (95% CI)	*p*-Value	HR (95% CI)	*p*-Value	HR (95% CI)	*p*-Value	HR (95% CI)	*p*-Value
NGS-MRD at post-HSCT-1m^a^									
Negative	91	1				1		1	
Positive	23	4.06 (1.64–10.05)	0.002			2.85 (1.39–5.83)	0.004	2.48 (1.14–5.42)	0.022
Disease state									
CR1	110	1				1		1	
CR2	4	1.98 (0.42–9.27)	0.384			2.27 (0.64–8.05)	0.204	3.22 (0.88–11.85)	0.078
Cohort									
Cohort #1	54			1					
Cohort #2	60			0.73 (0.22–2.41)	0.610				
Relationship									
Related	68			1					
Unrelated	46			3.06 (0.94–10.02)	0.064				
Sex match									
Female to male	20			1					
Others	94			0.26 (0.09–0.74)	0.012				

*CR1* first complete remission, *CR2* second complete remission, *HSCT* hematopoietic stem cell transplantation, *MRD* measurable residual disease, *n* number, *NGS* next-generation sequencing.

^a^NGS-MRD positive was defined by a failure of complete clearance of mutations (VAF cutoff of 0%).

We next classified patients into three groups according to pre-HSCT and post-HSCT-1m NGS-MRD status as follows (Fig. 2b): persistent MRD positivity group (*n* = 21), negative conversion of MRD positivity group (*n* = 30), and persistent MRD negativity group (*n* = 61). The risk of relapse was greatest in the persistent MRD positivity group and least in the persistent MRD negativity group (Fig. 2c). Survival analysis also showed significantly different DFS and OS among the three groups (Fig. 2d). This was supported by the results of the multivariate analysis (Supplementary Table S7). Of the two patients showing positive conversion of post-HSCT-1m NGS-MRD, one died of relapsed AML.

### Prognostic impact of persistent DTA or CHIP mutations

**Fig. 3:**
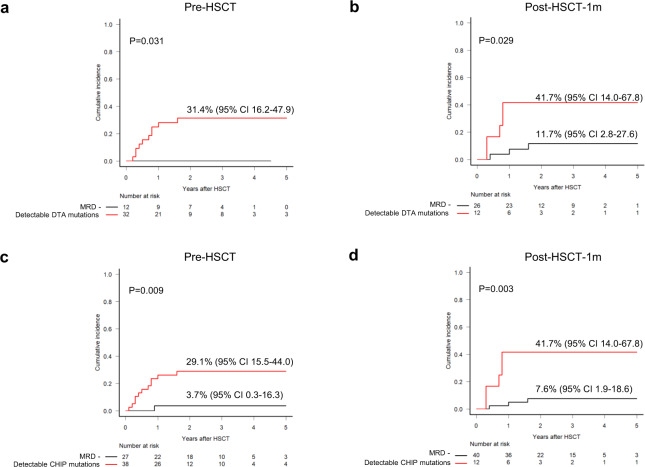
Prognostic impact of persistent DTA or CHIP mutations. Cumulative incidence of relapse according to detectable DTA (**a**, **b**) or CHIP (**c**, **d**) mutations at pre-HSCT (**a**, **c**) and post-HSCT-1m (**b**, **d**).

Among the 45 patients with DTA mutations at diagnosis, 32 (71%) and 12 (30%; 12/40) had persistent DTA mutations at pre-HSCT and post-HSCT-1m, respectively. Patients with detectable pre-HSCT or post-HSCT-1m DTA mutations had significantly higher CIR than those without detectable mutations (Fig. 3a, b). Among the 67 patients with CHIP mutations (including DTA mutations and *IDH2*, *IDH1*, *SF3B1*, *SRSF2*, *JAK2*, and *CBL*) at diagnosis, 57% (38/67) and 21% (12/56) had persistent CHIP mutations at pre-HSCT and post-HSCT-1m, respectively. Patients with detectable pre-HSCT or post-HSCT-1m CHIP mutations had significantly higher CIR than those without detectable mutations (Fig. 3c, d). The high CIR in patients with detectable DTA or CHIP mutations at post-HSCT-1m rather than at pre-HSCT translated into inferior survival (Supplementary Fig. S5).

### Effects of conditioning intensity on the prognostic value of NGS-MRD detection at each time point

**Fig. 4:**
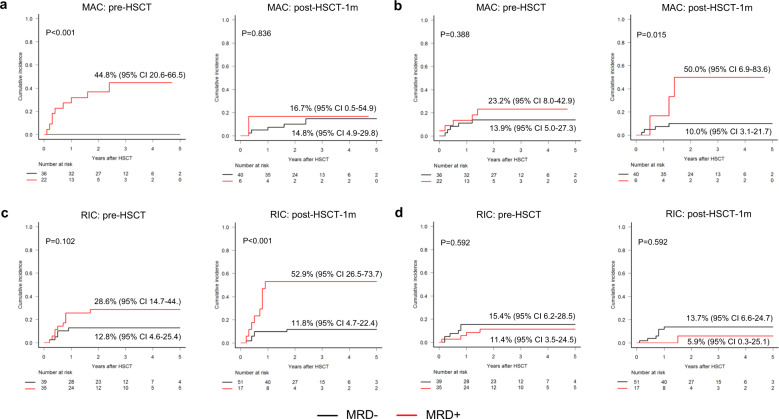
Effects of conditioning intensity on the prognostic value of NGS-MRD detection. Cumulative incidence of relapse (**a**, **c**) and non-relapse mortality (**b**, **d**) by NGS-MRD status at pre-HSCT and post-HSCT-1m according to conditioning intensity (myeloablative (MAC) and reduced-intensity conditioning (RIC)).

Given the significant differences in patient age and transplant-related characteristics (Supplementary Table S8) and the different degrees of dependence of transplant outcomes on graft-versus-leukemia effects according to conditioning intensity, we evaluated the impact of NGS-MRD at each time point according to conditioning intensity. In patients who received myeloablative conditioning (MAC, *n* = 58), pre-HSCT NGS-MRD detection was significantly associated with post-transplant relapse (Fig. 4a). However, there was no difference in relapse according to post-HSCT-1m NGS-MRD status. This may be partially attributable to the higher rate of NRM in NGS-MRD-positive patients than that in NGS-MRD-negative patients (Fig. 4b). In contrast, in patients who received reduced-intensity conditioning (RIC, *n* = 74), post-HSCT-1m NGS-MRD detection was significantly associated with post-transplant relapse, while there was no difference in relapse according to pre-HSCT NGS-MRD status (Fig. 4c). There was no difference in NRM according to NGS-MRD status at each time point in the RIC group (Fig. 4d). Consequently, in the MAC group, survival was significantly worse in the pre-HSCT NGS-MRD-positive patients than that in the NGS-MRD-negative patients. In the RIC group, post-HSCT-1m NGS-MRD-positive patients had worse survival than the NGS-MRD-negative patients (Supplementary Fig. S6).

### Clonal dynamics of mutations including later clearance and evolution after transplantation

We found that 67% (*n* = 38) and 52% (*n* = 12) of NGS-MRD-positive patients at pre-HSCT and post-HSCT-1m did not experience relapse, respectively (Supplementary Table S9). Among the 38 patients who were NGS-MRD positive at pre-HSCT, 23 (60.5%) converted to being NGS-MRD negative at post-HSCT-1m. The negative conversion rate did not significantly differ according to conditioning intensity (MAC vs. RIC: 58% vs. 65%). Of the 12 (28.9%) patients with persistent mutations at post-HSCT-1m, 7 (58%) had DTA mutations. We performed an NGS-MRD assay on BM samples taken 3 months after transplantation in 11 of these 12 patients. Ten patients became NGS-MRD negative, whereas one patient still had a persistent *DNMT3A* mutation (Fig. 1c).

BM samples at relapse were available in 17 patients (Supplementary Table S10). Most (16/17, 94%) of these patients had some or all of the same mutations at the time of both diagnosis and relapse. Longitudinal tracking revealed the appearance of detectable mutations at 2 or 3 months before relapse in three patients (#87, Fig. 1d; #89, Fig. 1e; #116, Fig. 1f). In addition, one patient (#90) with a *KRAS* mutation at initial diagnosis showed a *DNMT3A* mutation at post-HSCT-1m, which was thought to be of donor origin (Fig. 1g). The VAF of the *DNMT3A* mutation was markedly decreased at relapse, while three clonal mutations of the *FLT3*, *NRAS*, and *PTPN* genes had evolved at 29 months post-transplant.

## Discussion

We evaluated prognostic value of NGS-MRD assay in AML patients who underwent allo-HSCT at CR in two independent prospective cohorts. NGS-MRD detection has a prognostic value at both pre-HSCT and post-HSCT-1m, in each cohort, irrespective of mutation type, including DTA or CHIP mutations. Notably, we demonstrated that the prognostic impact of detectable mutations at each time point depended on the conditioning intensity and provided evidence for the benefit of serial NGS-MRD monitoring after allo-HSCT.

There is limited evidence on the prognostic value of dynamic changes of mutational clones detected by NGS-MRD assay in the setting of allo-HSCT in AML. In this study, mutational dynamics by NGS-MRD assay before and after transplantation showed a profound decrease in VAFs, but a relatively high persistence of DTA and CHIP mutations. However, most remaining pre-HSCT mutations, even DTA and CHIP mutations, disappeared after allo-HSCT. Any persistent mutations at pre-HSCT and post-HSCT-1m were significantly associated with post-transplant relapse and worse survival. Moreover, changes in MRD status from pre-HSCT to post-HSCT-1m enabled further identification of patients at higher risk for relapse and worse survival. These investigations including dynamic changes in NGS-MRD status are distinct from previous reports for the NGS-MRD assay in the setting of allo-HSCT, which contained no post-transplantation data, suggesting prognostic value of NGS-MRD at pre-HSCT. One study emphasized the prognostic value of post-transplant NGS-MRD (at 21 days after allo-HSCT) rather NGS-MRD at pre-HSCT^10^. Given those discordant data and limits of previous studies, such as retrospective nature of smaller cohorts data^7,11,13^ or the use of PB than BM at single time point^11,14^, the reliability of our data was supported by consistent results in two independent cohorts and use of BM for NGS-MRD assay.

Persistent DTA mutations are considered to be due to clonal hematopoiesis rather than residual leukemia. They have limited prognostic value after high-dose induction treatment^8,9,12^. Given the discordant findings on the role of persistent DTA mutations at pre-HSCT and the scarcity of information on the role of such mutations at post-HSCT in previous studies^10,11,13,14^, our data based on NGS-MRD detection clearly demonstrated the prognostic impact of persistent mutations in any gene (DTA or CHIP) at both pre-HSCT and post-HSCT-1m. Our findings suggest that these mutations are reliable MRD markers of post-transplant relapse. Allo-HSCT is a therapeutic approach to changing a patient’s hematopoietic system with donor tissue. Thus, any mutation, even one associated with clonal hematopoiesis, is expected to disappear if AML is cured. This idea is supported by the eventual clearance of persistent DTA and CHIP mutations in patients who never relapsed in our study.

The prognostic impact of the conditioning intensity on NGS-MRD detection was addressed in a phase III trial (BMT CTN 0901) that compared between MAC and RIC. The results of that trial showed that pre-transplant NGS-MRD detection in PB is associated with post-transplant relapse in patients who undergo RIC, rather than in those who undergo MAC^14^. However, the trial did not include post-transplant NGS-MRD detection data. As such, the findings need to be validated as a limited number of genes (*n* = 13) were sequenced from pre-HSCT blood DNA, with no data at diagnosis or post-HSCT. In addition, the CIR of the RIC group (47% at 1 year) in the trial was higher than that in other randomized phase III trials (17–30%)^20,21^. The current study, which sequenced a broader array of 67 genes from BM DNA at multiple time points during the peri-transplant period, demonstrated that persistent pre-HSCT mutations were associated with post-transplant relapse in patients who received MAC rather than in those who received RIC. Meanwhile, persistent post-HSCT-1m mutations were associated with post-transplant relapse in patients who received RIC rather than in those who received MAC. The limited impact of persistent post-HSCT-1m mutations might be biased by the high NRM in MRD-positive patients. A recent study on patients who received mostly MAC showed the significance of NGS-MRD at 21 days after HSCT^10^. Meanwhile, the prognostic value of NGS-MRD clearly differed according to time point (better for post-HSCT-1m than that for pre-HSCT) in the RIC group. The reliability of these findings is supported by the lack of difference in NRM rate according to MRD status in our study and the similarity between the CIR (20%) in this study and that of the RIC groups in previous randomized phase III trials^20,21^. These results suggest that the prognostic impact of NGS-MRD at pre-HSCT depends on the conditioning intensity in the opposite manner to that shown in the BMT CTN 0901 study^14^. Later time points appeared to be more reliable for NGS-MRD detection in the RIC group, which was more susceptible to graft-versus-leukemia effects than the MAC group. Further studies are needed to identify the precise effect of conditioning intensity on NGS-MRD results at different time points, using prospective cohorts of patients who are evenly distributed between the MAC and RIC groups.

We used conventional NGS-MRD and found that the most valuable VAF cutoff was 0% at both pre-HSCT and post-HSCT. At this cutoff, sensitivity was improved due to exclusion of mutations with low read depth, high background error rate, and allelic imbalance^18^. Using the NGS-MRD assay, we demonstrated the later clearance of persistent mutations after allo-HSCT, indicating a graft-versus-leukemia effect. Of note, we found that the NGS-MRD assay enables the detection of mutations before an overt relapse. Moreover, longitudinal analyses of relapsed samples revealed various conditions including different responses of mutations to treatment, mutational selection after treatment, and evolution of mutations during the peri-transplant period, thus increasing the utility of NGS-MRD. Interestingly, we were able to schematize donor-originated clonal hematopoiesis in detail, which could be discriminated from donor cell leukemia because it appeared just after allo-HSCT and disappeared during relapse. Taken together, these data provide evidence for the validity of serial NGS-MRD monitoring after allo-HSCT, although the technique needs to be upgraded with improved sequencing methods with higher sensitivity and a minimal error rate^22,23^.

In conclusion, persistent mutations at both pre-HSCT and post-HSCT-1m were associated with high risks of relapse and mortality regardless of mutation type, including DTA and CHIP. The optimal time point of NGS-MRD assessment depended on the conditioning intensity (pre-HSCT for MAC and post-HSCT-1m for RIC). Serial NGS-MRD monitoring after transplantation is a feasible way to compensate for the limited sensitivity and specificity of conventional NGS. The usefulness of NGS-MRD monitoring will facilitate trials investigating the feasibility of MRD-driven decision-making for risk-adapted approaches to reducing relapse in AML.

## Supplementary information

Supplementary materials
