# Supplementary material for: Prognostic value of measurable residual disease monitoring by next-generation sequencing before and after allogeneic hematopoietic cell transplantation in acute myeloid leukemia
Source: Blood Cancer J. 2021 Jun 4;11(6):109. doi: 10.1038/s41408-021-00500-9 (PMC8178334; doi:10.1038/s41408-021-00500-9)

Supplementary Information

Prognostic value of measurable residual disease monitoring by next-generation sequencing before and after allogeneic hematopoietic cell transplantation in acute myeloid leukemia

Index

1. Supplementary Materials and Methods
2. Supplementary Tables
3. Supplementary Figures

1. Supplementary Materials and Methods

(A) Validation of SM Acute leukemia panel and analytic algorithm

The Validation of NGS using SM Acute leukemia panel was performed according to the Guidelines for Validation of Next-Generation Sequencing–Based Oncology Panels(1) and The Standards and Guidelines for Validating Next-Generation Sequencing Bioinformatics Pipelines.(2) The analytical performance of SM Acute leukemia panel was evaluated in the previous study.(3) Annotated variants were classified into four tiers according to the Standards and Guidelines by the Association for Molecular Pathology.(4) All the variants with >0.01 minor allele frequency were filtered out based on public database including dbSNP (https://www.ncbi.nlm.nih.gov/snp/), genome aggregation database (gnomAD, https://gnomad.broadinstitute.org/), as well as an ethnic-specific Korean Variant Archive for a reference database of genetic variations in the Korean population (KOVA, http://kobic.re.kr /kova/). The variants, reported more than three times in AML in the Catalogue Of Somatic Mutations In Cancer (COSMIC, https://cancer.sanger.ac.uk/cosmic) were included. Among detected novel mutations, loss-of function mutations including nonsense, frameshift, or splicing mutations were included.

(B) QC matrices in clinical samples

Across all time points, the means of on-target reads, depth of on-target regions, and uniformity were 99.4%, 2406×, and 96.9%, respectively. Details of quality control matrices are summarized in Supplementary Table S3.

(C) NGS-MRD analysis

For NGS-MRD analysis, we selected trackable somatic mutations specific for each patient. We carefully inspected the mutations and determined a residual variant allele fraction (% VAF) through dividing mutant sequencing reads by total sequencing reads. To improve detection sensitivity, we excluded mutations with low read depth, high background error rate, and allelic imbalance when analyzed MRD.(1) All of the mutations were manually verified using the Integrative Genomic Viewer. Background error was calculated for each mutation and reached a mean and SD of 0.01% and 0.04%, respectively.

In addition, persisting hot spot mutations including *KRAS* codon 12 (G12D), *NRAS* codon 12 (G12D) and 13 (G13D) were confirmed by droplet digital PCR using QX200 ddPCR system and QuantaSoft Software (Bio-Rad, Hercules, CA) according to manufacturers' recommendation. Presence of *DNMT3A* codon 882 (R882H) mutation was also confirmed by real-time PCR as described by the previous study.(5)

References

1. Lee JM, Kim YJ, Park SS, Han E, Kim M, Kim Y. Simultaneous Monitoring of Mutation and Chimerism Using Next-Generation Sequencing in Myelodysplastic Syndrome. *J Clin Med* 2019;**8**(12) doi 10.3390/jcm8122077.
2. Roy S, Coldren C, Karunamurthy A, Kip NS, Klee EW, Lincoln SE*, et al.* Standards and Guidelines for Validating Next-Generation Sequencing Bioinformatics Pipelines: A Joint Recommendation of the Association for Molecular Pathology and the College of American Pathologists. *The Journal of Molecular Diagnostics* 2018;**20**(1):4-27 doi https://doi.org/10.1016/j.jmoldx.2017.11.003.
3. Park J, Kim HS, Lee J-M, Jung J, Kang D, Choi H*, et al.* Analytical and Potential Clinical Performance of Oncomine Myeloid Research Assay for Myeloid Neoplasms. *Molecular diagnosis & therapy* 2020;**24**(5):579-92 doi 10.1007/s40291-020-00484-5.
4. Li MM, Datto M, Duncavage EJ, Kulkarni S, Lindeman NI, Roy S*, et al.* Standards and Guidelines for the Interpretation and Reporting of Sequence Variants in Cancer: A Joint Consensus Recommendation of the Association for Molecular Pathology, American Society of Clinical Oncology, and College of American Pathologists. *J Mol Diagn* 2017;**19**(1):4-23 doi 10.1016/j.jmoldx.2016.10.002.
5. Park DJ, Kwon A, Cho B-S, Kim H-J, Hwang K-A, Kim M*, et al.* Characteristics of DNMT3A mutations in acute myeloid leukemia. *Blood Res* 2020;**55**(1):17-26 doi 10.5045/br.2020.55.1.17.

2. Supplementary Tables

Supplementary Table S1. Comparison of characteristics between cohort #1 and cohort #2.

Variables	Cohort #1 (n=63)	Cohort #2 (n=69)	*p*
NGS-MRD status at pre-HSCT, n (%)*			0.029
Negative	42 (67)	33 (48)	
Positive	21 (33)	36 (52)	
NGS-MRD status at post-HSCT-1m, n (%)*			0.147
Negative	40 (74)	51 (85)	
Positive	14 (26)	9 (15)	
Age at transplantation, years, n (%)			0.387
Median (range)	46 (19-69)	49 (19-74)	
Age group, n (%)			0.028
< 60	56 (89)	51 (74)	
≥ 60	7 (11)	18 (26)	
Sex, n (%)			0.276
Male	37 (59)	34 (49)	
Female	26 (41)	35 (51)	
AML type, n (%)			0.495
De novo	57 (91)	58 (84)	
Secondary	5 (8)	10 (14)	
Therapy-related	1 (2)	1 (2)	
WBC count, × 10^9/L at diagnosis, n (%)			0.271
Median (range)	16.02 (0.45-226.21)	10.56 (0.54-250.87)	
WBC group, × 10^9/L at diagnosis, n (%)			0.335
< 50	51 (81)	51 (74)	
≥ 50	12 (19)	18 (26)	
Cytogenetic risk group, n (%)**			0.777
Favorable	10 (16)	11 (16)	
Intermediate	40 (63)	47 (68)	
Adverse	13 (21)	11 (16)	
2017 ELN risk group, n (%)			0.291
Favorable	19 (30)	28 (41)	
Intermediate	26 (41)	20 (29)	
Adverse	18 (29)	21 (30)	
Disease status at HSCT, n (%)			0.049
CR1	59 (94)	69 (100)	
CR2	4 (6)	0	
Donor type, n (%)			<0.001
Matched sibling	0	27 (39)	
Matched unrelated	36 (57)	16 (23)	
Haploidentical	27 (43)	26 (38)	
Related			<0.001
Related	27 (43)	53 (77)	
Unrelated	36 (57)	16 (23)	
HLA disparity, n (%)			0.545
Full matched	36 (57)	43 (62)	
Mismatch	27 (43)	26 (38)	
Stem cell source, n (%)			0.368
Peripheral blood	63 (100)	65 (94)	
Bone marrow	0	4 (6)	
Conditioning intensity, n (%)			0.416
Myeloablative	30 (48)	28 (41)	
Reduced-intensity	33 (52)	41 (59)	

GVHD prophylaxis			<0.001
Cyclosporine + MTX	0	27 (39)	
Tacrolimus + MTD	63 (100)	42 (61)	
ATG, total dose			<0.001
Not used	0	16 (23)	
2.5 mg/kg	36 (57)	17 (25)	
5.0 mg/kg	27 (43)	36 (52)	
HCT-CI before transplantation, n (%)			0.333
0-2	46 (73)	45 (65)	
>2	17 (27)	24 (35)	
Sex match, n (%)			0.156
Female to male	13 (21)	8 (12)	
Others	50 (79)	61 (88)	

Abbreviations: AML, acute myeloid leukemia; ATG, anti-thymocyte globulin; CR1, first complete remission; CR2, second complete remission; ELN, European LeukemiaNet; GVHD, graft-versus-host disease; HCT-CI, hematopoietic cell transplant-comorbidity index; HSCT, hematopoietic stem cell transplantation; MRD, measurable residual disease; MTX, methotrexate; n, number; NGS, next-generation sequencing; WBC, white blood cells

∗ NGS-MRD positive was defined by a failure of complete clearance of mutations (VAF cutoff of 0%).

∗∗ Cytogenetic risk group was defined by refinement of cytogenetic classification by the United Kingdom Medical Research Council trials (Grimwade D, Hills RK, Moorman AV, et al. Refinement of cytogenetic classification in acute myeloid leukemia: determination of prognostic significance of rare recurring chromosomal abnormalities among 5876 younger adult patients treated in the United Kingdom Medical Research Council trials. Blood. 2010;116(3):354-365).

Supplementary Table S2. Gene targets of SM acute leukemia panel

Pathway	Gene
Chromatin/cohesin	*ASXL1, BCOR, EP300, EZH2, RAD21, SETD2, SMC1A, SMC3, STAG2*
DNA methylation	*DNMT3A, IDH1, IDH2, TET2*
RTK pathway	*BRAF, FLT3, KIT, KRAS, NF1, NRAS, PTPN11*
RNA splicing	*SF3B1, SRSF2, U2AF1, ZRSR2*
Transcription factor	*CEBPA, ETV6, GATA2, IKZF1, NOTCH1, RUNX1*
Nucleophosmin	*NPM1*
Tumor suppressor	*PHF6, TP53, WT1*
Others	*ANKRD26, BRINP3, CALR, CBL, CDKN2A, CDKN2B, CHEK1, CREBBP, CSF3R, DDX41, EGFR, ERG, FBXW7, GATA1, GATA3, HNRNPK, IL7R, JAK1, JAK2, JAK3, KMT2A, MPL, MYC, NOTCH3, NT5C2, PAX5, PDGFRA, PRAME, PTEN, RB1, SETBP1, SH2B3, TERT*

Abbreviations: RTK, receptor tyrosine kinase

Supplementary Table S3. Quality control matrices in all samples

**Parameters**	**Mean**	**95% Confidence interval**	**Remark**
Usable reads (%)	61	60.6 to 61.7	Per batch
Total number of bases ≧ AQ20 (bp)	536,201,214	526,252,917 to 546,149,511	Per batch
Total reads	2,727,288	2,690,512 to 2,794,064	Per Sample
Mapped reads	2,743,500	2,691,597 to 2,795,403	Per Sample
On-target reads (%)	99.4	99.3 to 99.4	Per Sample
Mean read length (bp)	211	211 to 211	Per Sample
Mean depth of on-target regions (X)	2,406	2,361 to 2,451	Per Sample
Uniformity of base coverage (%)	96.9	96.8 to 97	Per Sample

Supplementary Table S4. Rate of mutation clearance on the basis of genes and affected molecular pathways

Gene	Initial		Pre-HSCT		Post-HSCT-1m	
	No. of Mutations	No. of Patients	%	(No./Total No.)	%	(No./Total No.)
ASXL1	9	9	56	(5/9)	67	(2/3)
BCOR	12	11	75	(9/12)	100	(10/10)
CEBPA	34	27	91	(31/34)	96	(23/27)
CSF3R	8	6	100	(8/8)	100	(7/7)
DDX41	5	5	60	(3/5)	100	(5/5)
DNMT3A	28	26	21	(6/28)	79	(19/24)
EZH2	9	6	89	(8/9)	83	(5/6)
FLT3	22	22	95	(21/22)	100	(20/20)
GATA2	11	8	82	(9/11)	100	(10/10)
IDH1	10	10	90	(9/10)	100	(8/8)
IDH2	14	14	64	(9//14)	92	(11/12)
JAK3	5	3	100	(5/5)	100	(5/5)
KIT	19	14	100	(19/19)	93	(14/15)
KRAS	16	16	88	(14/16)	93	(13/14)
MYC	7	7	100	(7/7)	100	(7/7)
NPM1	28	28	89	(25/28)	96	(23/24)
NRAS	26	23	88	(23/26)	95	(19/20)
PTPN11	6	6	83	(5/6)	100	(6/6)
RAD21	7	6	71	(5/7)	86	(6/7)
RUNX1	13	10	77	(10/13)	82	(9/11)
SETD2	7	6	86	(6/7)	100	(8/8)
SF3B1	6	5	33	(2/6)	100	(5/5)
SMC1A	6	6	67	(4/6)	80	(4/5)
SMC3	5	5	80	(4/5)	82	(5/5)
TET2	20	17	30	(6/20)	80	(16/20)
WT1	9	9	100	(9/9)	100	(6/6)
Pathway						
DTA	57	45	30	(17/57)	79	(37/47)
CHIP	98	67	46	(45/98)	86	(70/81)
Chromatin/cohesin	62	50	74	(45/61)	90	(44/49)
DNA methylation	72	55	42	(30/72)	84	(54/64)
RTK pathway	93	63	91	(85/93)	96	(75/78)
RNA splicing	12	10	58	(7/12)	100	(10/10)
Transcription factor	63	42	87	(55/63)	92	(47/51)
Tumor suppressor	15	15	87	(13/15)	100	(11/11)

Abbreviations: HSCT, hematopoietic stem cell transplantation; CHIP, clonal hematopoiesis of indeterminate potential; RTK, receptor tyrosine kinase

DTA: *ASXL1, DNMT3A, TET2*
CHIP: DTA and *CBL, IDH2, JAK2, IDH1, SF3B1, SRSF2*
Chromatin/cohesin: *ASXL1, BCOR, EP300, EZH2, RAD21, SETD2, SMC1A, SMC3, STAG2*
DNA methylation: *DNMT3A, IDH1, IDH2, TET2*
RTK pathway: *BRAF, FLT3*, *KIT, KRAS, NF1, NRAS, PTPN11*
RNA splicing: *SF3B1, SRSF2, U2AF1, ZRSR2*
Transcription factor: *CEBPA, ETV6, GATA2, IKZF1, NOTCH1, RUNX1*
Tumor suppressor: *PHF6, TP53, WT1*

Supplementary Table S5. Factors affecting survival outcomes (Univariate analysis)

Univariate variables	n	Cumulative incidence of relapse		Cumulative incidence of non-relapse mortality		Disease-free survival		Overall survival	
		HR (95% CI)	P value	HR (95% CI)	P value	HR (95% CI)	P value	HR (95% CI)	P value
NGS-MRD status at pre-HSCT*									
Negative	75	1		1		1		1	
Positive	57	5.91 (2.20-15.86)	<0.001	1.25 (0.52-3.02)	0.618	2.70 (1.46-4.99)	0.002	2.24 (1.19-4.22)	0.013
NGS-MRD status at post-HSCT-1m*									
Negative	91	1		1		1		1	
Positive	23	4.47 (1.89-10.57)	0.001	1.89 (0.60-5.96)	0.278	3.20 (1.63-6.27)	0.001	2.96 (1.44-6.07)	0.003
Cohort									
Cohort #1	63	1		1		1		1	
Cohort #2	69	1.16 (0.51-2.62)	0.720	0.27 (0.10-0.74)	0.011	0.62 (0.34-1.13)	0.117	0.54 (0.29-1.02)	0.059
Age	132	1.00 (0.97-1.03)	0.989	1.03 (1.00-1.07)	0.097	1.01 (0.99-1.04)	0.255	1.013 (0.99-1.04)	0.278
Age group									
< 60 years	107	1		1		1		1	
≥ 60 years	25	0.84 (0.29-2.45)	0.744	0.50 (0.52-3.90)	0.498	0.83 (0.52-2.26)	0.830	1.10 (0.50-2.38)	0.819
Sex									
Male	71	1		1		1		1	
Female	61	0.51 (0.22-1.20)	0.125	0.69 (0.28-1.68)	0.410	0.59 (0.32-1.09)	0.090	0.62 (0.33-1.18)	0.142
WBC at diagnosis	132	1.00 (1.00-1.00)	0.318	1.00 (1.00-1.00)	0.631	1.00 (1.00-1.00)	0.679	1.00 (1.00-1.00)	0.572
WBC group at diagnosis									
< 50 × 10^9/L	102	1		1		1		1	
≥ 50 × 10^9/L	30	0.62 (0.21-1.81)	0.378	1.31 (0.50-3.42)	0.577	0.90 (0.45-1.83)	0.780	0.91 (0.44-1.92)	0.810
AML type									
De novo	115	1		1		1		1	
Secondary and therapy-related	17	1.21 (0.41-3.53)	0.731	0.69 (0.16-2.95)	0.612	0.96 (0.41-2.28)	0.931	0.88 (0.34-2.24)	0.783
Cytogenetic risk group**			0.625		0.230		0.683		0.803
Favorable	21	1		1		1		1	
Intermediate	87	0.85 (0.28-2.59)	0.776	4.22 (0.56-31.71)	0.162	1.52 (0.59-3.92)	0.383	1.37 (0.53-3.55)	0.517
Adverse	24	1.37 (0.39-4.84)	0.630	1.86 (0.17-20.51)	0.612	1.47 (0.48-4.50)	0.499	1.23 (0.39-3.88)	0.721
2017 ELN risk group			0.255		0.161		0.345		0.562
Favorable	47	1		1		1		1	
Intermediate	46	0.79 (0.27-2.27)	0.658	2.95 (0.94-9.26)	0.064	1.50 (0.72-3.14)	0.281	1.26 (0.58-2.72)	0.561
Adverse	39	1.75 (0.69-4.43)	0.240	1.72 (0.46-6.42)	0.417	1.74 (0.81-3.71)	0.155	1.53 (0.71-3.30)	0.283
Disease state									
CR1	128	1		1		1		1	
CR2	4	4.56 (1.06-19.62)	0.042	2.77 (0.37-20.95)	0.324	3.75 (1.15-12.22)	0.028	4.69 (1.43-15.41)	0.011
Donor type			0.887		0.056		0.393		0.243
Matched sibling	27	1		1		1		1	
Matched unrelated	52	0.77 (0.27-2.22)	0.630	3.79 (0.86-16.80)	0.079	1.52 (0.68-3.44)	0.311	1.94 (0.78-4.83)	0.156
Haploidentical	53	0.90 (0.33-2.47)	0.832	1.36 (0.26-7.00)	0.715	1.01 (0.43-2.39)	0.977	1.23 (0.47-3.21)	0.667
Related									
Related	80	1		1		1		1	
Unrelated	52	0.83 (0.35-1.93)	0.661	3.08 (1.23-7.72)	0.017	1.51 (0.84-2.73)	0.172	1.68 (0.90-3.12)	0.101
Stem cell source									
Peripheral blood	128	1		1		1		1	
Bone marrow	4	1.43 (0.19-10.60)	0.727	0.05 (0-5315.13)	0.608	0.77 (0.11-5.59)	0.796	0.82 (0.11-5.97)	0.844
HLA disparity									
Full matched	79	1		1		1		1	
Mismatched	53	1.05 (0.47-2.36)	0.909	0.49 (0.18-1.35)	0.169	0.76 (0.41-1.42)	0.390	0.77 (0.40-1.48)	0.441

Sex match									
Female to male	21	1		1		1		1	
Others	111	0.87 (0.30-2.54)	0.795	0.32 (0.13-0.79)	0.014	0.52 (0.26-1.02)	0.058	0.56 (0.26-1.17)	0.120
HCT-CI									
0-2	91	1		1		1		1	
>2	41	1.37 (0.60-3.13)	0.455	0.75 (0.27-2.07)	0.583	1.06 (0.56-2.00)	0.856	1.21 (0.63-2.31)	0.571
Conditioning intensity									
Myeloablative	58	1		1		1		1	
Reduced-intensity	74	1.29 (0.56-2.94)	0.549	0.79 (0.33-1.89)	0.591	1.03 (0.56-1.86)	0.936	0.95 (0.51-1.77)	0.876
Conditioning intensity			0.815		0.356		0.554		0.685
Myeloablative	58	1		1		1		1	
Reduced-toxicity	46	1.23 (0.49-3.11)	0.657	0.51 (0.16-1.61)	0.249	0.85 (0.42-1.73)	0.658	0.82 (0.39-1.70)	0.590
Reduced-intensity	28	1.38 (0.49-3.89)	0.542	1.25 (0.45-3.45)	0.664	1.31 (0.64-2.71)	0.461	1.19 (0.55-2.57)	0.668
GVHD prophylaxis									
Cyclosporin + MTX	27	1		1		1		1	
Tacrolimus + MTX	105	0.84 (0.33-2.11)	0.704	2.53 (0.59-10.90)	0.213	1.26 (0.59-2.71)	0.556	1.57 (0.66-3.74)	0.310
ATG, total dose			0.289		0.117		0.051		0.117
Not used	16	1		1		1		1	
2.5 mg/kg	53	4.58 (0.60-35.21)	0.144	2.36 (0.53-10.57)	0.261	3.10 (0.93-10.29)	0.065	2.65 (0.79-8.90)	0.114
5.0 mg/kg	63	3.19 (0.41-24.74)	0.266	0.89 (0.18-4.43)	0.891	1.67 (0.49-5.68)	0.416	1.52 (0.44-5.23)	0.503

Abbreviations: AML, acute myeloid leukemia; ATG, anti-thymocyte globulin; CR1, first complete remission; CR2, second complete remission; ELN, European LeukemiaNet; GVHD, graft-versus-host disease; HCT-CI, hematopoietic cell transplant-comorbidity index; HSCT, hematopoietic stem cell transplantation; MRD, measurable residual disease; MTX, methotrexate; n, number; NGS, next-generation sequencing; WBC, white blood cells

∗ NGS-MRD positive was defined by a failure of complete clearance of mutations (VAF cutoff of 0%).

∗∗ Cytogenetic risk group was defined by refinement of cytogenetic classification by the United Kingdom Medical Research Council trials (Grimwade D, Hills RK, Moorman AV, et al. Refinement of cytogenetic classification in acute myeloid leukemia: determination of prognostic significance of rare recurring chromosomal abnormalities among 5876 younger adult patients treated in the United Kingdom Medical Research Council trials. Blood. 2010;116(3):354-365).

Supplementary Table S6. Predicting performance of NGS-MRD detection for post-transplant relapse according to various cutoffs at each time point

Various cutoffs at each time point of MRD assessment	MRD positive patents	Relapsed patients among MRD positive patients	Positive predictive value	Sensitivity	MRD negative patents	Non-relapsed patients among MRD negative patients	Negative predictive value	Specificity
Pre-HSCT (n=132)								
VAF 0%	57	19	33.3%	79.2%	75	70	93.3%	64.8%
VAF 0.2%	49	16	32.6%		83	75	90.3%	
VAF 1.0%	39	12	30.7%		93	81	87.0%	
VAF 2.0%	33	11	33.3%		99	86	86.9%	
VAF 5.0%	25	8	32.0%		107	91	85.0%	
Post-HSCT-1m (n=114)								
VAF 0%	23	10	43.5%	47.6%	91	80	87.9%	86.0%
VAF 0.2%	12	4	33.3%		102	85	83.3%	
VAF 1.0%	5	2	40.0%		109	90	82.6%	
VAF 2.0%	4	2	50.0%		110	91	82.7%	
VAF 5.0%	1	1	100%		113	93	82.3%	

Abbreviations: HSCT, hematopoietic stem cell transplantation; MRD, measurable residual disease; n, number; NGS, next-generation sequencing; VAF, variant allele frequency

Supplementary Table S7. Multivariate analysis for changes in NGS-MRD status between pre-HSCT and post-HSCT-1m.

Variables (n=114)	n	Relapse		Disease-free survival		Overall survival	
		HR (95% CI)	P value	HR (95% CI)	P value	HR (95% CI)	P value
Changes of NGS-MRD status*			0.005		0.005		0.047
From negative to negative	61	1		1		1	
From positive to negative	30	4.03 (1.18-13.80)	0.026	2.26 (0.98-5.22)	0.056	1.81 (0.75-4.38)	0.186
From positive to positive	21	7.57 (2.22-25.79)	0.001	4.03 (1.72-9.42)	0.001	3.10 (1.26-7.66)	0.014
Disease state							
CR1	108	1		1		1	
CR2	4	2.03 (0.42-9.74)	0.001	2.27 (0.64-8.05)	0.204	3.22 (0.86-12.09)	0.083

Abbreviations: CR1, first complete remission; CR2, second complete remission; HSCT, hematopoietic stem cell transplantation; MRD, measurable residual disease; n, number; NGS, next-generation sequencing

* NGS-MRD positive was defined by a failure of complete clearance of mutations (VAF cutoff of 0%).

Supplementary Table S8. Comparison of clinical characteristics between MAC and RIC (n=132)

Variables	Myeloablative conditioning (n=58)	Reduced-intensity conditioning (n=74)	*p*
NGS-MRD status at pre-HSCT, n (%)*			0.281
Negative	36 (62)	39 (53)	
Positive	22 (38)	35 (47)	
NGS-MRD status at post-HSCT-1m, n (%)*			0.119
Negative	40 (87)	51 (75)	
Positive	6 (13)	17 (25)	
Cohort			0.416
Cohort #1	30 (52)	33 (45)	
Cohort #2	28 (48)	41 (55)	
Age at transplantation, years, n (%)			<0.001
Median (range)	38 (19-59)	55 (19-74)	
Age group, n (%)			<0.001
< 60	58 (100)	49 (66)	
≥ 60	0	25 (34)	
Sex, n (%)			0.674
Male	30 (52)	41 (55)	
Female	28 (48)	33 (45)	
AML type, n (%)			0.265
De novo	50 (86)	65 (88)	
Secondary	6 (10)	9 (12)	
Therapy-related	2 (3)	0	
WBC count, $\times 10^9$/L at diagnosis, n (%)			0.919
Median (range)	15.7 (0.45-250.9)	10.7 (0.54-266.2)	
WBC group, $\times 10^9$/L at diagnosis, n (%)			0.732
< 50	44 (76)	58 (78)	
≥ 50	14 (24)	16 (22)	
Cytogenetic risk group, n (%)**			0.227
Favorable	10 (17)	11 (15)	
Intermediate	34 (59)	53 (72)	
Adverse	14 (24)	10 (14)	
2017 ELN risk group, n (%)			0.076
Favorable	17 (29)	30 (41)	
Intermediate	18 (31)	28 (38)	
Adverse	23 (40)	16 (22)	
Disease status at HSCT, n (%)			0.130
CR1	58 (100)	70 (95)	
CR2	0	4 (5)	
Donor type, n (%)			<0.001
Matched sibling	20 (34)	7 (9)	
Matched unrelated	38 (66)	14 (19)	
Haploidentical	0	53 (72)	
Related			<0.001
Related	20 (34)	55 (57)	
Unrelated	38 (66)	18 (19)	
HLA disparity, n (%)			<0.001
Full matched	58 (100)	21 (28)	
Mismatch	0	53 (72)	
Stem cell source, n (%)			0.035
Peripheral blood	54 (93)	74 (100)	
Bone marrow	4 (7)	0	
GVHD prophylaxis			<0.001
Cyclosporine + MTX	20 (34)	7 (9)	
Tacrolimus + MTD	38 (66)	67 (91)	
ATG, total dose			<0.001
Not used	13 (22)	3 (4)	
2.5 mg/kg	43 (74)	10 (13)	
5.0 mg/kg	2 (3)	61 (82)	

HCT-CI before transplantation, n (%)			0.995
0-2	40 (69)	51 (69)	
>2	18 (31)	23 (31)	
Sex match, n (%)			0.286
Female to male	7 (12)	14 (19)	
Others	51 (88)	60 (81)	

Abbreviations: AML, acute myeloid leukemia; ATG, anti-thymocyte globulin; CR1, first complete remission; CR2, second complete remission; ELN, European LeukemiaNet; GVHD, graft-versus-host disease; HCT-CI, hematopoietic cell transplant-comorbidity index; HSCT, hematopoietic stem cell transplantation; MRD, measurable residual disease; MTX, methotrexate; n, number; NGS, next-generation sequencing; WBC, white blood cells

* NGS-MRD positive was defined by a failure of complete clearance of mutations (VAF cutoff of 0%).

** Cytogenetic risk group was defined by refinement of cytogenetic classification by the United Kingdom Medical Research Council trials (Grimwade D, Hills RK, Moorman AV, et al. Refinement of cytogenetic classification in acute myeloid leukemia: determination of prognostic significance of rare recurring chromosomal abnormalities among 5876 younger adult patients treated in the United Kingdom Medical Research Council trials. Blood. 2010;116(3):354-365).

Supplementary Table S9. Patients characteristics and mutations at each time point of patients with positive MRD but without relapse (n=38)

UNP	Age	ELN	Conditioning	aGVHD	cGVHD	Non-relapse deaths	Survival from HSCT (days)	Mutations at initial diagnosis	Persistent mutations at pre-HSCT	Persistent mutations at post-HSCT-1m	Persistent mutations at post-HSCT-3m
72	64	Favorable	RIC	no	severe	yes	551	*CEBPA/FLT3/NPM1/NRAS/PTPN11/RAD21*(2)*/TET2*	*RAD21/TET2*	*RAD21/TET2*	None
71	63	Favorable	RIC	no	no	no	999	*DNMT3A/NPM1/TET2*	*DNMT3A/TET2*	*TET2*	None
114	61	Adverse	RIC	grade II	moderate	no	557	*TET2*(2)	*TET2*(2)	*TET2*	None
8	47	Intermediate	MAC	grade II	moderate	yes	500	*SETPB1/SMC1A*	*SMC1A*	*SMC1A*	None
103	68	Adverse	RIC	no	no	no	651	*CEBPA/EZH2*(2)*/RUNX1*(2)*/TET2*(2)	*RUNX1*(2)*/TET2*(2)	*RUNX1*(2)	None
15	56	Intermediate	RIC	no	mild	no	2087	*BCOR/KRAS/NPM1*	*NPM1*	*KRAS*	None
125	43	Intermediate	RIC	no	no	no	744	*DNMT3A/FLT3/WT1*	*DNMT3A*	*DNMT3A*	None
58	21	Favorable	MAC	no	no	no	1609	*CEBPA*(2)*/RAD21*	*CEBPA*	*CEBPA*	None
21	34	Favorable	MAC	grade II	severe	no	1685	*CEBPA*(2)*/GATA2*(2)	*CEBPA*	*CEBPA*	None
37	45	Adverse	RIC	no	mild	no	2262	*ASXL1/CEBPA/DNMT3A*(2)*/IDH2*	*ASXL1*	*ASXL1*	None
136	56	Adverse	MAC	grade I	mild	yes	453	*ASXL1/DNMT3A/IDH1*	*ASXL1/DNMT3A/IDH2/RUNX1*	*DNMT3A*	*DNMT3A*
16	53	Intermediate	RIC	grade II	mild	no	2270	*GATA2/IDH1/NPM1/NRAS/SMC1A*	*IDH1/NRAS*	NA	None
32	59	Adverse	MAC	no	no	yes	12	*DNMT3A/JAK2*	*DNMT3A/JAK2*	NA	NA
22	55	Favorable	RIC	grade I	moderate	no	2146	*DNMT3A/FLT3/NPM1/NRAS*	*DNMT3A*	NA	NA
51	55	Intermediate	MAC	grade III	severe	yes	185	*DNMT3A/IDH1*	*DNMT3A*	*DNMT3A*	NA
146	35	Adverse	MAC	grade IV	no	yes	188	*ASXL1/CEBPA/SETBP1/U2AF1*	*ASXL1/SETBP1/U2AF1*	None	
12	61	Intermediate	RIC	no	no	yes	52	*DNMT3A/NPM1/SMC1A*	*DNMT3A*	None	
130	57	Intermediate	MAC	grade III	no	yes	90	*DDX41/NOTCH3*	*DDX41*	None	
115	59	Intermediate	RIC	grade II	no	yes	308	*SETD2/SMC1A*	*SETD2/SMC1A*	None	
95	46	Favorable	RIC	grade II	severe	yes	371	*CBL/DNMT3A/IDH1/NPM1/SMC1A*	*CBL/DNMT3A*	None	
111	55	Favorable	RIC	grade III	severe	no	604	*DNMT3A/FLT3/NPM1/TET2*	*DNMT3A/TET2*	None	
106	58	Favorable	RIC	grade II	mild	no	612	*NPM1/RAD21*	*NPM1/RAD21*	None	
107	62	Intermediate	RIC	no	mild	no	519	*DDX41/EZH2/SH2B3*	*DDX41*	None	
79	39	Adverse	RIC	no	no	no	654	*NRAS/RUNX1/WT1*	*NRAS*	None	
134	65	Favorable	RIC	no	moderate	no	984	*NPM1/PTPN11/SMC3/TET2*	*TET2*	None	
81	68	Favorable	RIC	grade I	mild	no	1177	*FLT3/NPM1/PTPN11/SF3B1/SMC3/TET2*	*SF3B1/TET2*	None	
53	41	Intermediate	RIC	grade II	mild	no	2228	*DNMT3A/IDH1/STAG2*	*DNMT3A*	None	
113	19	Favorable	MAC	no	no	no	592	*NRAS/TP53*	*NRAS*	None	
135	54	Intermediate	MAC	no	no	no	964	*FLT3/SF3B1/WT1*	*SF3B1*	None	
139	55	Intermediate	RIC	no	no	no	889	*FBXW7/NOTCH1/PHF6*	*FBXW7*	None	
102	60	Adverse	MAC	no	mild	no	651	*IDH2*	*IDH2*	None	
117	70	Intermediate	RIC	no	mild	no	575	*BRAF/TET2*	*BRAF/TET2*	None	
124	62	Adverse	RIC	no	mild	no	492	*ASXL1/BCOR/DNMT3A/IDH2/ RUNX1*	*BCOR/DNMT3A/IDH2/ RUNX1*	None	
131	46	Intermediate	RIC	no	mild	no	1027	*IDH2/STAG2*	*IDH2*	None	
143	53	Favorable	RIC	grade I	mild	no	557	*ASXL1/NRAS/TET2/TP53*	*TET2/TP53*	None	
132	57	Adverse	MAC	grade II	mild	no	1063	*DNMT3A/FLT3/PHF6/RUNX1/TET2*	*DNMT3A/TET2*	None	
75	39	Favorable	RIC	no	moderate	no	977	*DNMT3A/NPM1*	*DNMT3A*	None	
144	38	Adverse	MAC	no	severe	no	517	*BCOR*(2)*/GATA2/KRAS/SF3B1*(2)	*BCOR*(2)*/GATA2/KRAS/SF3B1*(2)	None	

Abbreviations: aGVHD, acute graft-versus-host disease; cGVHD, chronic graft-versus-host disease; European LeukemiaNet; HSCT, hematopoietic stem cell transplantation; MAC, myeloablative conditioning; MRD, measurable residual disease; RIC, reduced-intensity conditioning; UNP, unit number of patients

Supplementary Table S10. Patients characteristics and comparison of mutations at initial and relapse (n=17)

UNP	Age	ELN	Conditioning	aGVHD	cGVHD	Disease-free survival from HSCT (days)	Mutations at initial diagnosis	Mutations at relapse
98	42	Adverse	RIC	no	no	696	*JAK1/NRAS/RAD21/SH2B3*	*NRAS/RAD21/SH2B3*
49	52	Favorable	MAC	grade I	mild	573	*DNMT3A/FLT3/GATA2*(2)*/NPM1*	*DNMT3A/FLT3/GATA2*(2)*/NPM1*
84	37	Intermediate	MAC	grade II	no	136	*DNMT3A*	*DNMT3A*
66	26	Adverse	MAC	no	no	96	*EP300/JAK3*(3)*/SETD2/RUNX1/*	*EP300/JAK1/JAK3*(2)*/SETD2*
89	54	Intermediate	MAC	grade I	no	375	*DNMT3A/IDH2*	*DNMT3A/FLT3/IDH2/NRAS*
91	45	Adverse	RIC	no	no	91	*KRAS/NRAS(2)/SETD2/TP53/*	*NRAS/SETD2/TP53*
90	58	Intermediate	MAC	no	severe	868	*KRAS*	*FLT3/NRAS/PTPN11*
68	59	Adverse	RIC	no	no	138	*PTPN11/RUNX1*	*PTPN1/RUNX1*
88	49	Intermediate	RIC	no	mild	142	*FLT3/KRAS*	*KRAS*
35	39	Favorable	RIC	no	no	96	*FLT3/KIT/TET2*(2)	*TET2*(2)
70	36	Favorable	MAC	no	no	74	*DNMT3A/FLT3/KRAS/NPM1*	*DNMT3A/FLT3/KRAS/NPM1/WT1*
19	37	Adverse	MAC	no	no	92	*ASXL1/CEBPA/EZH2/IDH2*	*ASXL1/CEBPA/IDH2*
87	45	Adverse	MAC	no	no	243	*ASXL1/BCOR/CEBPA/TP53*	*ASXL1/BCOR/TP53*
116	28	Adverse	RIC	grade II	mild	275	*BCOR/CSF3R/DNMT3A/IDH2/RUNX1*	*BCOR/DNMT3A/IDH2/RUNX1*
65	29	Favorable	RIC	grade I	no	67	*EZH2/KIT*	*EZH2/KIT*
129	59	Intermediate	RIC	no	no	256	*BCOR/DNMT3A*(2)*/IDH1/KRAS*	*ASXL1/DNMT3A/IDH1*
28	54	Adverse	RIC	no	no	284	*DNMT3A*	*DNMT3A*

Abbreviations: aGVHD, acute graft-versus-host disease; cGVHD, chronic graft-versus-host disease; European LeukemiaNet; HSCT, hematopoietic stem cell transplantation; MAC, myeloablative conditioning; MRD, measurable residual disease; RIC, reduced-intensity conditioning; UNP, unit number of patients

Yellow highlight indicates clonal mutations disappear at relapse.

Blue highlight indicates newly evolving mutations.

3. Supplementary Figure

Supplementary Fig. S1. Trial profile

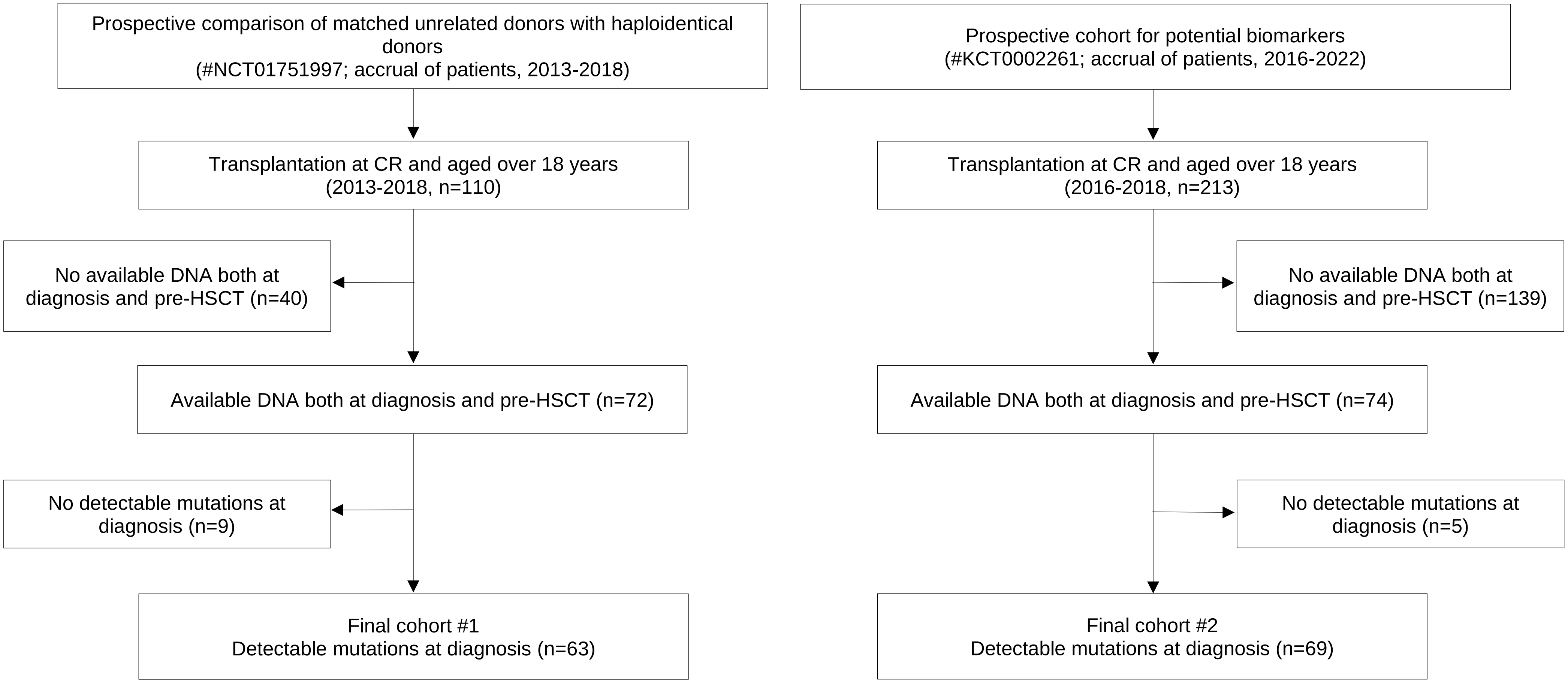

Supplementary Fig. S2. Genetic landscapes for all patients

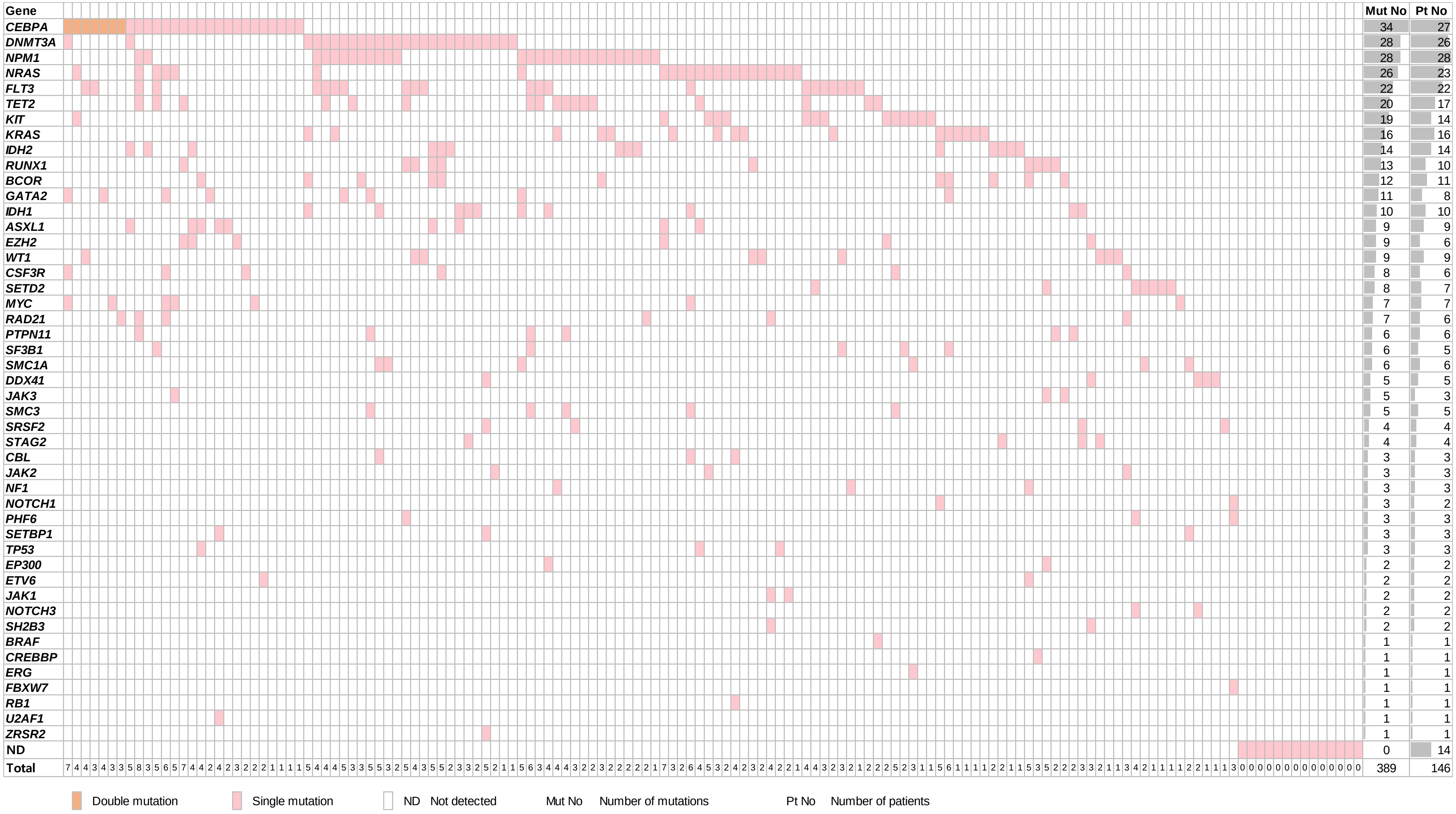

Supplementary Fig. S3. Cumulative incidence of relapse and overall survival by NGS-MRD status at pre-HSCT (A, B, E, and F) and post-HSCT-1m (C, D, G, and H) in cohort #1 (A-D) and cohort #2 (E-H). Cumulative incidence of relapse (A, C, E, and G) and overall survival (B, D, F and H)

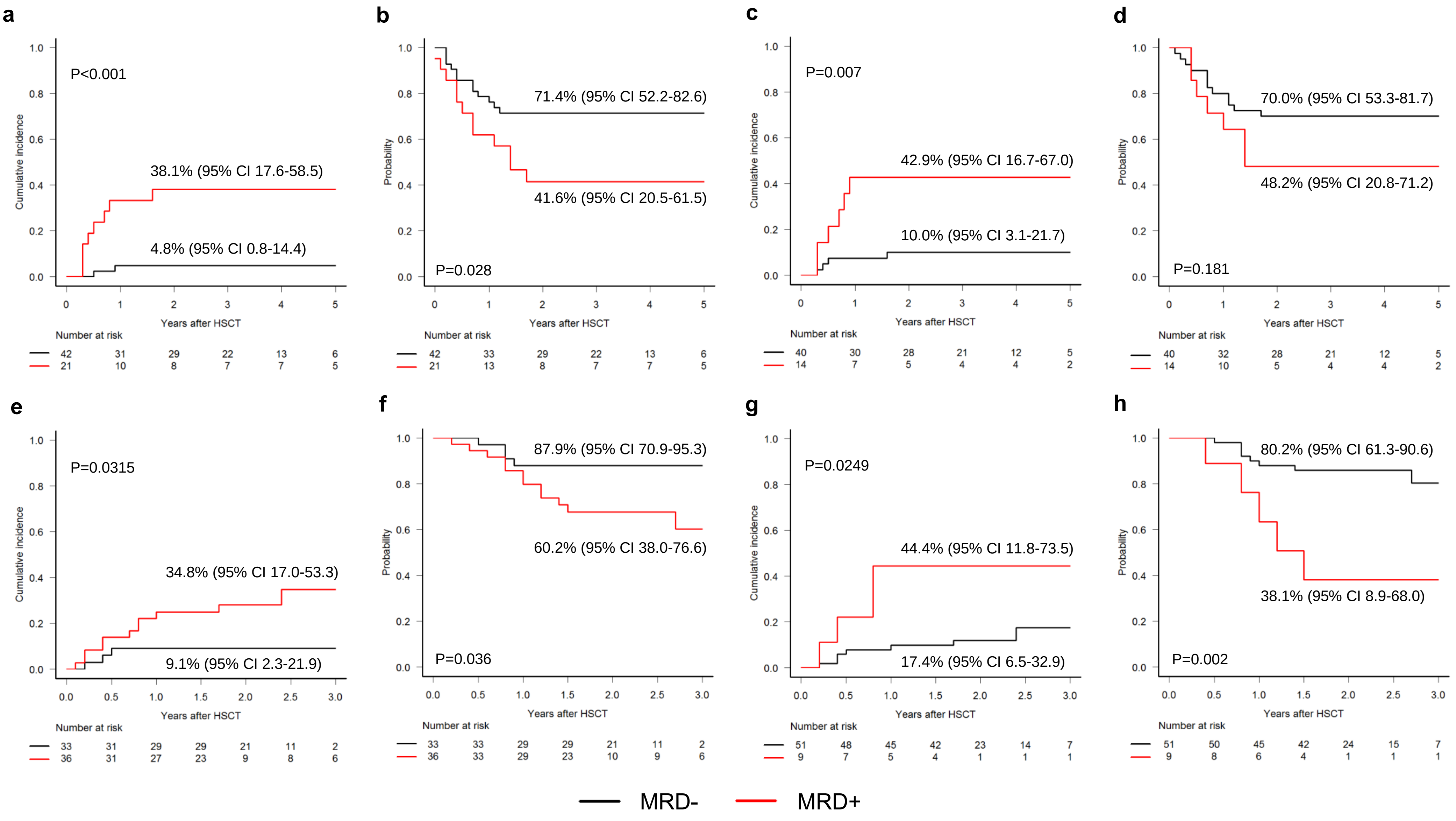

Supplementary Fig. S4. Cumulative incidence of relapse by various cutoffs of VAF at pre-HSCT (A) and post-HSCT-1m (B).

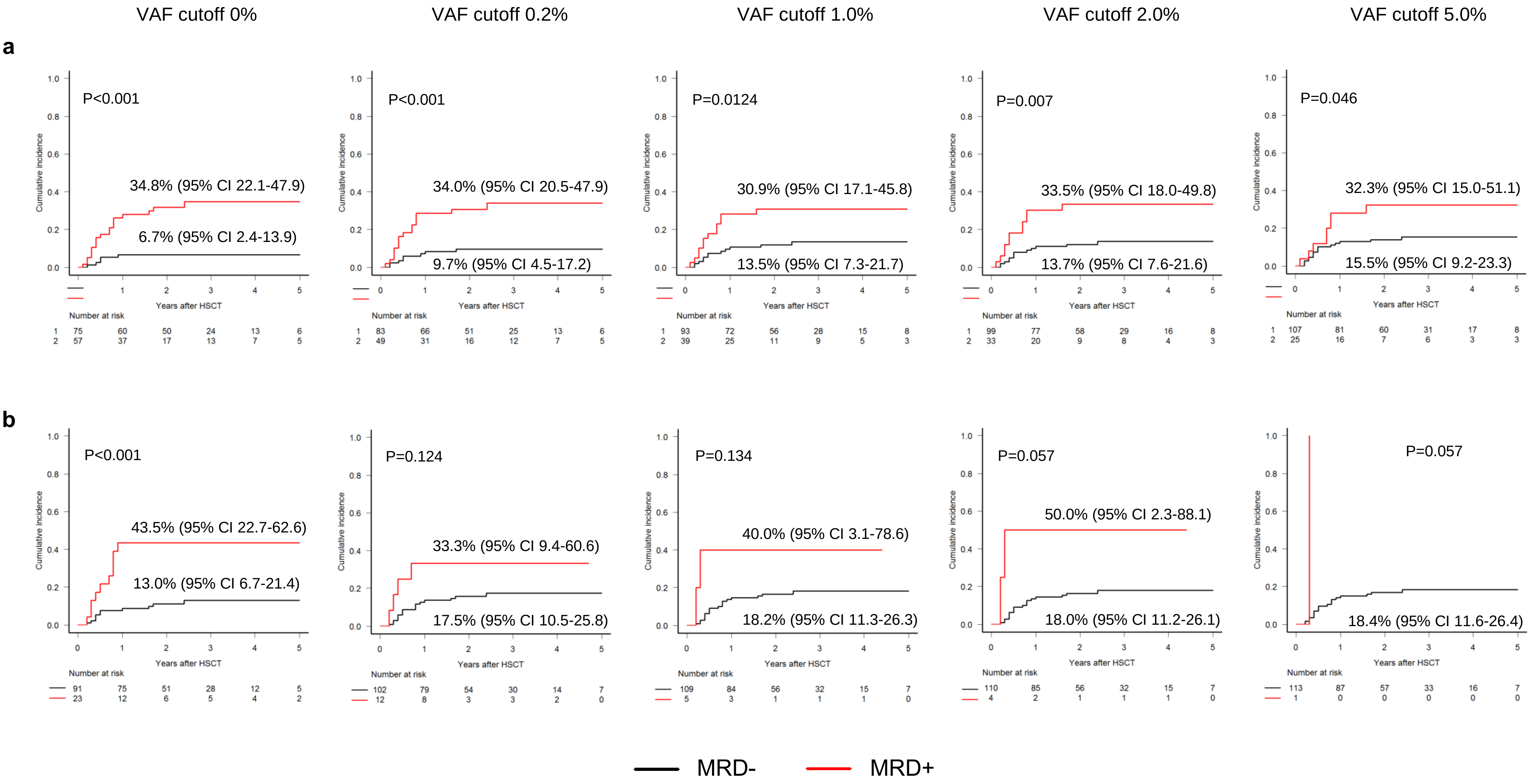

Supplementary Fig. S5. Cumulative incidence of disease-free survival (A and B) and overall survival (C and D) by detectable DTA (A and C) or CHIP (B and D)

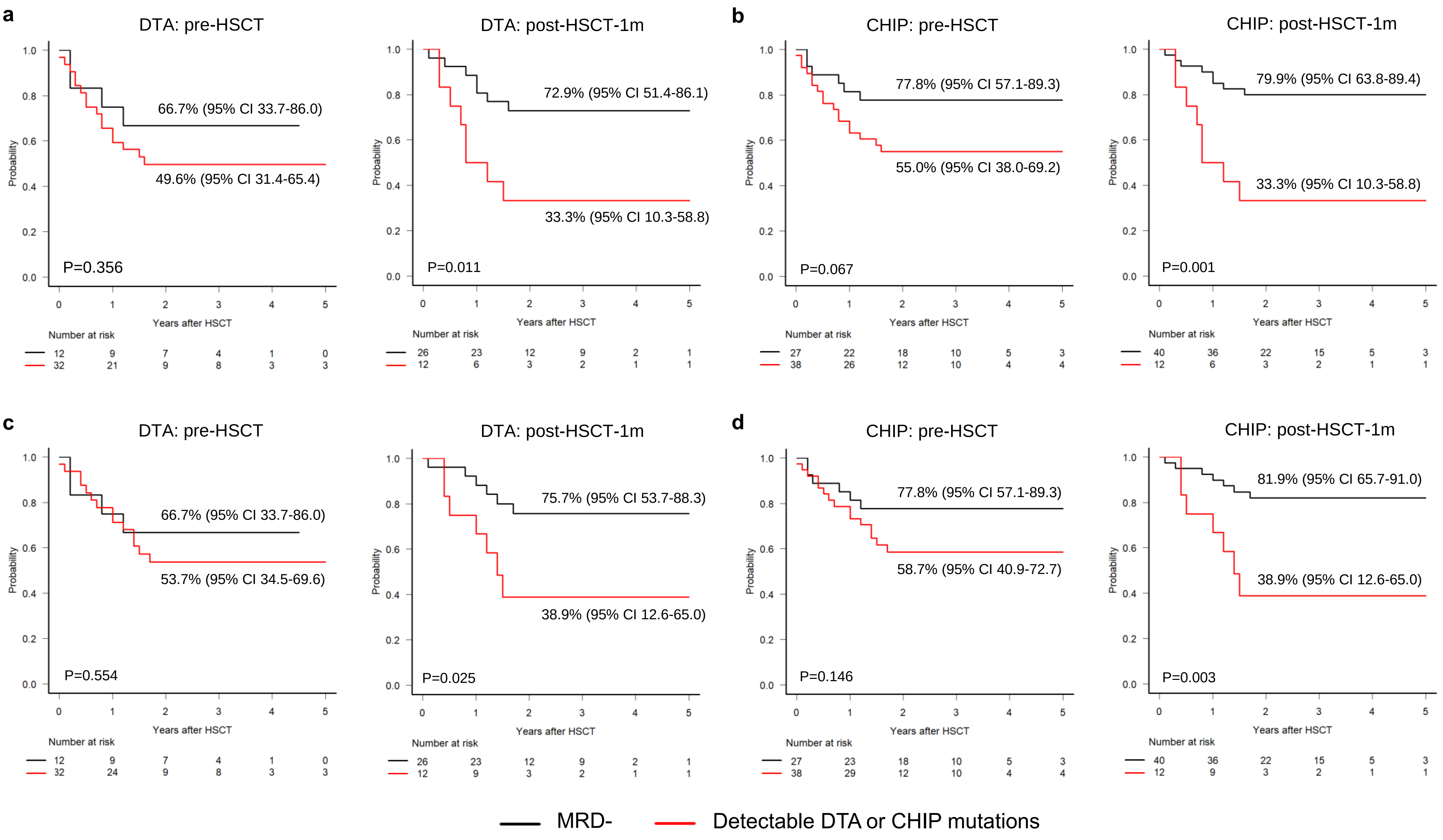

Supplementary Fig. S6. Cumulative incidence of disease-free survival (A and B) and overall survival (C and D) by NGS-MRD status in patients who received myeloablative (A and C) and reduced-intensity conditioning (B and D).

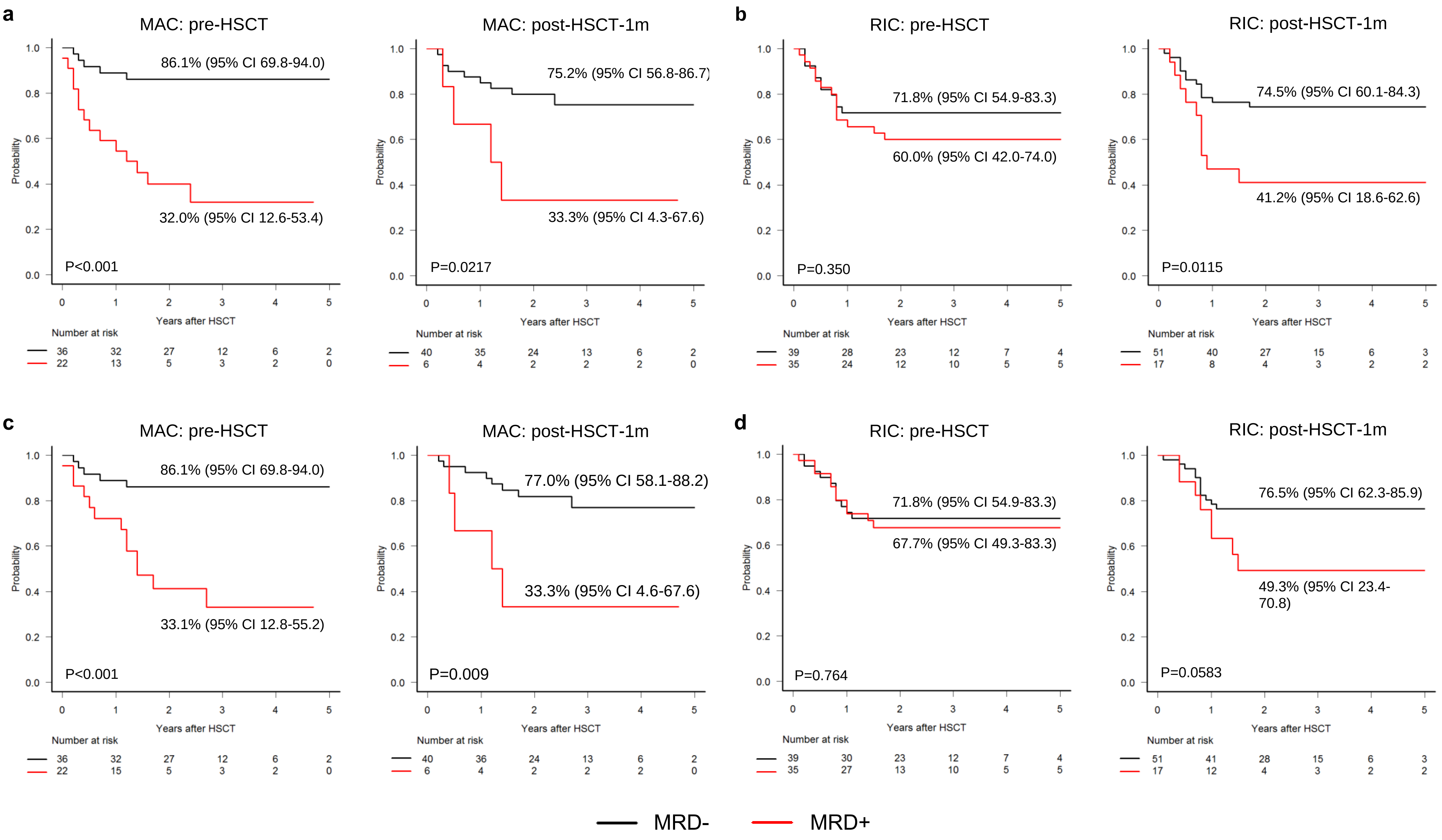

Supplement: Supplementary file 1 — Supplementary materials [file 41408_2021_500_MOESM1_ESM.pdf]
